# Adaptive laboratory evolution under acetic acid stress enhances the multistress tolerance and ethanol production efficiency of *Pichia kudriavzevii* from lignocellulosic biomass

**DOI:** 10.1038/s41598-023-48408-7

**Published:** 2023-11-28

**Authors:** Sureeporn Dolpatcha, Huynh Xuan Phong, Sudarat Thanonkeo, Preekamol Klanrit, Mamoru Yamada, Pornthap Thanonkeo

**Affiliations:** 1 Department of Biotechnology, Faculty of Technology, Khon Kaen University, Khon Kaen, 40002 Thailand; 2 Department of Microbial Biotechnology, Institute of Food and Biotechnology, Can Tho University, Can Tho, 900000 Vietnam; 3 Walai Rukhavej Botanical Research Institute, Mahasarakham University, Maha Sarakham, 44150 Thailand; 4 Fermentation Research Center for Value Added Agricultural Products (FerVAAPs), Faculty of Technology, Khon Kaen University, Khon Kaen, 40002 Thailand; 5 Department of Biological Chemistry, Faculty of Agriculture, Yamaguchi University, Yamaguchi, 753-8515 Japan; 6 Research Center for Thermotolerant Microbial Resources, Yamaguchi University, Yamaguchi, 753-8515 Japan

**Keywords:** Biotechnology, Microbiology

## Abstract

Second-generation bioethanol production using lignocellulosic biomass as feedstock requires a highly efficient multistress-tolerant yeast. This study aimed to develop a robust yeast strain of *P. kudriavzevii* via the adaptive laboratory evolution (ALE) technique. The parental strain of *P. kudriavzevii* was subjected to repetitive long-term cultivation in medium supplemented with a gradually increasing concentration of acetic acid, the major weak acid liberated during the lignocellulosic pretreatment process. Three evolved *P. kudriavzevii* strains, namely, PkAC-7, PkAC-8, and PkAC-9, obtained in this study exhibited significantly higher resistance toward multiple stressors, including heat, ethanol, osmotic stress, acetic acid, formic acid, furfural, 5-(hydroxymethyl) furfural (5-HMF), and vanillin. The fermentation efficiency of the evolved strains was also improved, yielding a higher ethanol concentration, productivity, and yield than the parental strain, using undetoxified sugarcane bagasse hydrolysate as feedstock. These findings provide evidence that ALE is a practical approach for increasing the multistress tolerance of *P. kudriavzevii* for stable and efficient second-generation bioethanol production from lignocellulosic biomass.

## Introduction

Ethanol is one of the biorenewable energies determined by the Ministry of Energy, Thailand, as part of the 15-year renewable energy development strategy (2008–2022). The target goal of bioethanol production and utilization is approximately 20% of the country's final energy consumption by 2022^1^. Approximately 95% of bioethanol utilized currently is produced via the biological fermentation process, and the primary feedstocks for bioethanol production in Thailand are sugarcane molasses and cassava. Since these feedstocks are also utilized in other industries, such as food, feed, and fine chemical production, competition of feedstocks for producing these products may occur, particularly when the feedstock demand is increased. The supply of feedstocks may need to be increased, but the feedstock price may also be high, which may affect the overall living cost and the country's overall economic growth. Thus, alternative feedstocks with high potential that are not being used in the food, feed, and fine chemical production industries can solve those problems.

Several economic crops are grown in Thailand, such as rice, corn, cassava, sugarcane, and pineapple, that generate a large number of byproducts, such as rice straw, corncobs, sugarcane bagasse, cassava pulp, and pineapple peel, respectively, after the manufacturing process. These byproducts are considered lignocellulosic materials, which can be used as feedstocks for second-generation bioethanol production. Based on chemical composition analysis, these raw materials comprise a large amount of cellulose and hemicellulose, accounting for approximately 30–50% and 20–30% by weight, respectively. For instance, rice straw contains approximately 30–40% cellulose and 20–30% hemicellulose, and corn cobs contain 40% cellulose and 20% hemicellulose on a dry basis^2,3^. Since these raw materials are in large quantity, inexpensive, and readily available in the country, they are considered high-potential feedstocks for domestic ethanol production.

The drawbacks of using lignocellulosic materials for bioethanol production include the complex pretreatment process, high-cost operation, and the generation of lignocellulosic inhibitors, such as acetic acid, furfural, 5-(hydroxymethyl) furfural (5-HMF), and phenolic acid, that inhibit the growth and metabolic activity of ethanol-producing microorganisms. To overcome these circumstances, several high-efficiency pretreatment methodologies that can reduce operation costs and the formation of inhibitors have been reported, such as steam explosion^4^, liquid hot water, dilute-acid hydrolysis^5^, the organosolv process^6^ and ammonia fiber explosion (AFEX)^7^.

Among lignocellulosic inhibitors, acetic acid (CH_3_COOH) is the most abundant weak acid, accounting for 5–10 g/L, depending on the type of feedstock and pretreatment process^8–10^. High concentrations of acetic acid inhibit the growth and ethanol production of several ethanologenic microorganisms, e.g., *Candida shehatae*, *Pichia stipitis*, *Saccharomyces cerevisiae*^11^, *Kluyveromyces marxianus*^12^ and *Zymomonas mobilis*^13,14^. Several strategies have been applied to minimize the inhibitory effect of acetic acid on microbial growth and ethanol production, for instance, the removal of acetic acid using sodium borohydride^15^ and activated carbon^16^, the application of dried air stripping^17^, or genetic engineering of microbial cells harboring the genes involved in acetic acid tolerance, such as *FPS1*^18^, *PHO13*^19^, and *HAA1*^20^.

Adaptive laboratory evolution (ALE) is one of the most promising strategies for improving microbial phenotypes or physiological characteristics through long-term cultivation^21^. ALE not only enables an understanding of the microbial evolution process under certain environmental conditions but also provides novel targets for creating microbial cell factories through metabolic engineering for bioproduction^22^. Compared with metabolic engineering, ALE allows the redirection of metabolism without considering the metabolic networks of the microbial cells. It has recently been widely applied for improving carbon source utilization^23,24^, enhancing the production efficiency of target biomolecules^25–27^, and creating stress-tolerant microorganisms toward thermal, ethanol, acetic acid, and lignocellulosic inhibitors^13,28–30^. Regarding the application of ALE for the improvement of yeast properties, most studies have focused on the conventional species *S. cerevisiae*^31–37^. Based on a literature review, only a few studies have reported the application of ALE to select robust strains of non-*Saccharomyces* yeasts, specifically *Pichia kudriavzevii*, one of the most thermotolerant yeasts extensively studied for bioethanol and other biomolecule production^38–40^.

It has previously been demonstrated that the damage induced by thermal or other stresses, such as ethanol, acetic acid, formic acid, phenolic acid, and furfural, shares specific common characteristics, e.g., leakage of the cell membrane, accumulation of reactive oxygen species (ROS), and denaturation of macromolecules, such as proteins, DNA or RNA, which subsequently trigger stress-responsive mechanisms to protect yeast cells from severe stress conditions. Furthermore, yeast cells that exhibit high resistance to one stress may also possess the ability to withstand other stress conditions^41,42^. Thus, the present study aims to improve the multistress tolerance capability of *P. kudriavzevii* toward lignocellulosic inhibitors through the ALE strategy using acetic acid as a selective pressure. The growth performance of the selected evolved strains under various stress conditions was determined. Furthermore, the ethanol fermentation efficiency of the selected evolved strains using sugarcane bagasse hydrolysate (SBH) without a detoxifying process was also evaluated.

## Materials and methods

### Chemicals and culture medium

The bacteriological grade of yeast extract, malt extract, and peptone was procured from TM Media (Titan Biotech Ltd., Delhi, India). Acetic acid, furfural, 5-HMF (analytical grade), vanillin, isopropanol, and ethanol (HPLC grade) were purchased from Sigma‒Aldrich, St. Louis, MO, USA. Glucose (analytical grade) was obtained from KemAusTM, New South Wales, Australia.

Yeast extract malt extract (YM) medium (3 g/L yeast extract, 3 g/L malt extract, 5 g/L peptone, and 10 g/L glucose) was used to cultivate yeast cells. After preparation, the medium was autoclaved at 121 °C and 15 psi for 15 min.

### Plant material

The sugarcane bagasse used in the present study was kindly provided by the sugar factory in Khon Kaen province, Thailand, with the permission of the Chief Executive Officer. After drying, the bagasse was pulverized into small pieces and kept in a plastic bag at room temperature at the Department of Biotechnology, Faculty of Technology, Khon Kaen University, with the code number KKUDB-SB-2022-01. All plant preparation methods followed the relevant guidelines in the Methods section.

The chemical compositions of the sugarcane bagasse, including cellulose, hemicellulose, and lignin, were determined using the standard method of the Association of Official Analytical Chemists^43^.

### Yeast strain and inoculum preparation

*Pichia kudriavzevii,* a thermotolerant yeast isolated from soil^39^, was used in this study. It was cultured in YM agar and maintained at 4 °C by subculturing every two months.

Yeast inoculum was prepared by transferring a loopful of yeast cells into 100 mL YM broth and incubated in a controlled incubator shaker (JSR, Gongju, Republic of Korea) at 35 °C with a shaking speed of 150 rpm for 24 h. Then, the activated yeast cells were transferred into fresh YM broth with an initial cell concentration of approximately 1 × 10^5^ cells/mL and subsequently incubated under the abovementioned conditions. After 12 h of incubation, the growing yeast cells were used as a starter culture or inoculum for subsequent experiments.

### Adaptive laboratory evolution (ALE) of *P. kudriavzevii* under acetic acid stress

Based on the preliminary study, acetic acid at a concentration of 7 g/L was a critical point for the growth of *P. kudriavzevii* since less than 10% of yeast cells survived under long-term cultivation in YM medium containing 7 g/L acetic acid at 35 °C. Thus, the ALE of *P. kudriavzevii* was commenced using acetic acid at 7 g/L, and the adaptation process was performed using a protocol described by Samappito et al.^13^ with some modifications. Three steps of the cultivation process were employed in this study. In the first step, a starter culture of yeast cells (1 × 10^6^ cells/mL) was inoculated into 50 mL YM broth containing 7 g/L acetic acid and incubated at 35°C and 150 rpm. After 48 h of incubation, the cells were transferred into fresh YM medium containing acetic acid at 7 g/L and repeatedly cultivated under the same conditions until the growth profile of the yeast was stable (cell number reached 1 × 10^8^ cells/mL). A total of 24 cycles was performed in the first step, and the resulting evolved yeast strain, designated PkAC-7, was obtained. In the second step, cells of PkAC-7 (1 × 10^6^ cells/mL) were inoculated into 50 mL YM broth containing 8 g/L acetic acid and incubated at 35°C and 150 rpm for 72 h. Then, cells were transferred into fresh YM medium containing 8 g/L acetic acid, and the cultivation process was repeated under the same conditions for 45 cycles. The evolved strain of yeast designated PkAC-8 was obtained in this step. Finally, the same experimental procedure as the second step was conducted using the evolved PkAC-8 as a starter culture and YM medium containing 9 g/L acetic acid as the selective pressure. The cultivation process was repeated for 53 cycles, and the evolved strain designated PkAC-9 was obtained. The parental and selected evolved strains of *P. kudriavzevii* were subjected to further studies.

### Stability test of *P. kudriavzevii* evolved strains

The YM media containing 7, 8, and 9 g/L acetic acid were prepared and used to cultivate *P. kudriavzevii* PkAC-7, PkAC-8, and PkAC-9, respectively. To test the stability of all *P. kudriavzevii* evolved strains to withstand a high concentration of acetic acid, starter cultures of the yeast cells were inoculated into 100 mL YM medium containing acetic acid as mentioned earlier with an initial yeast cell concentration of 1 × 10^6^ cells/mL. After incubation at 35 °C and 150 rpm for 48 h (PkAC-7) or 72 h (PkAC-8 and PkAC-9), cells were transferred into fresh YM medium without acetic acid supplementation and subsequently incubated as previously mentioned. The cultivation process was repeated at least 20 cycles for each strain of yeasts, and the yeast cell viability was determined.

The growth profile of *P. kudriavzevii* wild-type and evolved strains was determined by culturing yeast cells in YM medium containing 7, 8, and 9 g/L acetic acid and incubating at 35 °C and 150 rpm. The YM medium without acetic acid supplementation was used as a control condition. During cultivation, cells were randomly collected at certain time intervals, and the growth of yeast cells was determined using a haemacytometer (H-0004, Boeco, Germany) with methylene blue staining.

### Analysis of cell morphology using scanning electron microscopy (SEM)

The *P. kudriavzevii* parental and evolved strains were grown in YM broth at 35 °C and 150 rpm for 24 h. The cells were collected by filtration using 0.45 μm filter paper and dried at 60 °C for 24 h. The resulting cells were coated with gold by ion sputter (EMITECH model K500X, Kent, UK), and the morphology of the yeast cells was visualized using SEM (FEI, Helios NanoLab G3 CX, Australia).

### Characterization of cell growth under stress conditions

The inhibitory effects of heat, ethanol, osmotic, and lignocellulosic inhibitors, including acetic acid, formic acid, furfural, 5-HMF, and vanillin, on the growth of the *P. kudriavzevii* parental and evolved strains were determined using the procedure described by Pilap et al.^42^ and Gurdo et al.^44^, with some modifications. An initial cell concentration of 1 × 10^6^ cells/mL was used throughout this experiment. For heat stress, starter cultures of yeast cells were transferred into 100 mL YM medium and incubated at 35, 37, and 40 °C and 150 rpm for 48 h. Ethanol stress was evaluated by transferring yeast cells into 100 mL YM medium containing 0, 7, 10, and 13% (v/v) ethanol and incubated at 35 °C and 150 rpm for 48 h. Osmotic stress was assayed by inoculating yeast cells into 100 mL YM medium containing 0, 0.6, and 1.2 M sorbitol and incubated at 35 °C and 150 rpm for 12 h. For lignocellulosic inhibitor stress, different concentrations of acetic acid (0, 9, 12 g/L), formic acid (0, 1, 2 g/L), furfural (0, 1, 5 g/L), 5-HMF (0, 1, 3 g/L), and vanillin (0, 1, 2 g/L) were tested. The yeast cells were inoculated into 100 mL YM containing each inhibitor at different concentrations and incubated at 35 °C and 150 rpm for 48 h. The effect of cocktail inhibitors, including 9 g/L acetic acid, 2 g/L formic acid, 5 g/L furfural, 3 g/L 5-HMF, and 2 g/L vanillin, on the growth of yeast cells was also determined. After transferring yeast cells into 100 mL YM medium containing a cocktail inhibitor at different loadings (25, 50, 75, and 100% loading), the cultures were incubated at 35 °C and 150 rpm for 48 h. The viability of the yeast cells after the stress treatments was measured using a haemacytometer (H-0004, Boeco, Germany) with methylene blue staining.

### Preparation of sugarcane bagasse hydrolysate (SBH)

SBH was prepared following the method described by Sritrakul et al.^10^. Briefly, dried sugarcane bagasse (10% w/v solid loading) was transferred into 3% (v/v) sulfuric acid solution and soaked at room temperature overnight. The mixture was heated at 121 °C and 15 psi for 60 min. After pretreatment, the liquid fraction (or acid hydrolysate) was collected by filtration through a muslin cloth and directly used as feedstock for ethanol production without a detoxifying process. The solid fraction after acid pretreatment was collected after washing with running tap water, dried at 60 °C until constant weight, and then subjected to enzymatic hydrolysis. A sample of the acid-pretreated solid fraction (5% w/v solid loading) was soaked in 50 mM sodium citrate buffer (pH 4.8), and cellulase (Cellic® CTec2) at a concentration of 15 filter paper units (FPU)/g dry solid was added. The suspension mixture was incubated at 50 °C and 200 rpm for 24 h. The enzymatic reaction was stopped by incubating the sample mixture in boiling water for 5 min. After centrifugation at 8000 rpm for 10 min, the resulting enzymatic hydrolysate was collected and supplemented with acid hydrolysate for ethanol production. The concentrations of sugars and lignocellulosic inhibitors in the acid and enzymatic hydrolysates were determined using high-performance liquid chromatography (HPLC).

### Ethanol production from SBH by *P. kudriavzevii* parental and evolved strains

Batch ethanol fermentation from SBH by *P. kudriavzevii* parental and evolved strains was performed in a 250-mL Erlenmeyer flask. A starter culture of yeast cells was inoculated into 100 mL SBH supplemented with 1.5 g/L yeast extract and 3.0 g/L peptone with an initial cell concentration of approximately 1 × 10^7^ cells/mL. The flasks were incubated in a controlled incubator shaker (JSR, Gongju, Republic of Korea) at 35 °C and 150 rpm. Samples were randomly withdrawn at specific time intervals, and the ethanol and sugar concentrations in the fermentation broth were analyzed using gas chromatography (GC) and phenol–sulfuric acid method, respectively.

#### Analytical methods

The yeast growth as viable cells was determined using a hemocytometer (H-0004, Boeco, Germany) with methylene blue staining. Total sugar was measured by the phenol–sulfuric acid method^45^ using glucose as a standard. The ethanol concentration (P, g/L) was analyzed by GC (GC-14B, Shimadzu, Kyoto, Japan) using a protocol of Laopaiboon et al.^46^. The volumetric ethanol productivity (Qp, g/L h) and ethanol yield (Yp/s, g ethanol produced/g total sugar consumed) were calculated as described by Nuanpeng et al.^47^. All experiments were performed twice, each with two replications, and the results are expressed as the means ± standard deviations (SDs). Duncan’s multiple-range test (DMRT) was applied to analyze the mean differences between each treatment at a probability of *p* ≤ 0.05 using the SPSS program for Windows.

## Results and discussion

### Adaptive laboratory evolution (ALE) of *P. kudriavzevii* under acetic acid stress

**Figure 1:**
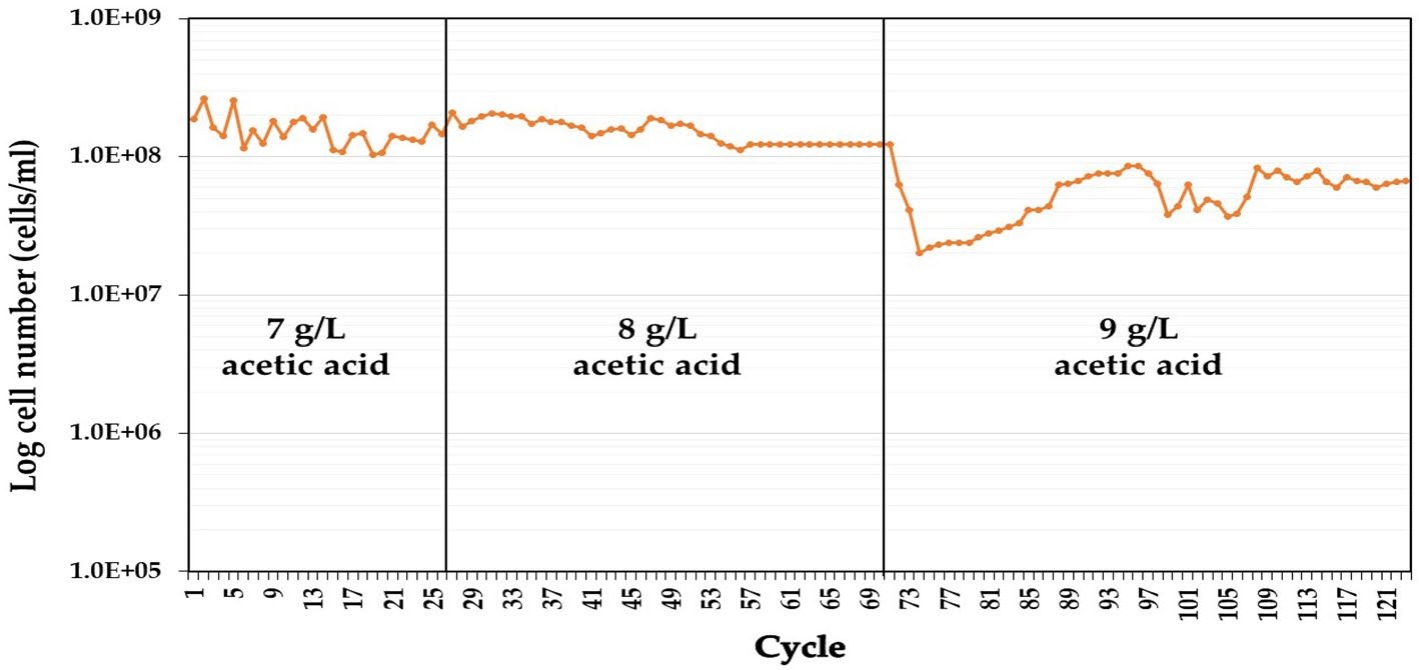
The growth profile of *P. kudriavzevii* after repeated long-term cultivation in YM medium containing acetic acid at 7, 8, and 9 g/L.

Three evolved strains of *P. kudriavzevii,* designated PkAC-7, PkAC-8, and PkAC-9, were obtained after long-term cultivation in YM medium containing acetic acid as a selective pressure. The growth profile of yeast cells during the ALE process is illustrated in Fig. 1. In the medium containing 7 g/L acetic acid, approximately 10^8^ cells/mL of yeast cells were reached at each cultivation cycle. Similarly, a constant number of yeast cells (approximately 10^8^ cells/mL) was also detected using YM medium containing 8 g/L acetic acid. However, a dramatic decrease in yeast cell numbers was found when the acetic acid concentration in the cultivation medium was shifted from 8 to 9 g/L, specifically during the first cultivation period. After long-term repeated cultivation for 53 cycles, a slight increase in yeast cell number and a constant value of approximately 10^8^ cells/mL was observed at the end of the cultivation period, suggesting that the evolved strains adapted and acquired tolerance capability to high concentrations of acetic acid. The results in the present study align with those reported in the literature. For instance, González-Ramos et al.^48^ and Gurdo et al.^44^ successfully developed acetic acid-tolerant strains of *S. cerevisiae* for ethanol production using ALE in medium supplemented with acetic acid. Another study by Zhang et al.^37^ also obtained *S. cerevisiae* yeast with very high thermotolerance after long-term adaptation in wheat straw hydrolysate at 35–42 °C. To the best of our knowledge, this is the first report to demonstrate the application of ALE using acetic acid as a selective pressure for selecting acetic acid-tolerant *P. kudriavzevii* strains for ethanol production.

The stability of all *P. kudriavzevii* evolved strains to withstand a high concentration of acetic acid was assessed by repeat cultivation in YM medium with and without acetic acid supplementation for 20 cycles at 35 °C, as described in the Materials and methods. The results revealed that all selected evolved strains exhibited high cell viability (more than 95%) under acetic acid stress. A comparative analysis of the growth of the parental and evolved strains was carried out using YM medium supplemented with acetic acid at different concentrations at 35 °C, and the growth profiles of the yeasts are illustrated in Supplementary Fig. S1. In YM medium without acetic acid supplementation (control treatment), the evolved strains, particularly PkAC-9 and PkAC-8, but not PkAC-7, exhibited significantly higher specific growth rates than the parental strain. The differences in the specific growth rates of the yeast strains were significantly distinguished when the medium was supplemented with 7, 8, and 9 g/L acetic acid. Among the evolved strains, *P. kudriavzevii* PkAC-9 displayed the highest specific growth rate under all stress conditions (Table 1). These findings suggested that repetitive long-term cultivation with gradually increasing acetic acid concentrations in the culture medium dramatically improved the growth performance of *P. kudriavzevii* at 35 °C under acetic acid stress.

**Table 1:** The specific growth rate of the *P. kudriavzevii* parental and evolved strains grown at 35 °C in YM medium supplemented with acetic acid at different concentrations.

Yeast strain	Specific growth rate (h^−1^) at different acetic acid concentrations			
	0 g/L (control)	7.0 g/L	8.0 g/L	9.0 g/L
Parental	0.93 ± 0.02^c^	0.09 ± 0.01^d^	0.08 ± 0.02^c^	0.06 ± 0.01^c^
PkAC-7	0.95 ± 0.01^bc^	0.24 ± 0.02^c^	0.11 ± 0.01^b^	0.09 ± 0.02^b^
PkAC-8	0.98 ± 0.02^b^	0.53 ± 0.01^b^	0.29 ± 0.02^a^	0.28 ± 0.02^a^
PkAC-9	1.09 ± 0.01^a^	0.67 ± 0.02^a^	0.30 ± 0.02^a^	0.30 ± 0.01^a^

Means ± standard deviations (SDs) with the same letters in the same column are not significantly different at *p* ≤ 0.05 based on DMRT analysis.

### Cell morphology of the *P. kudriavzevii* parental and evolved strains

**Figure 2:**
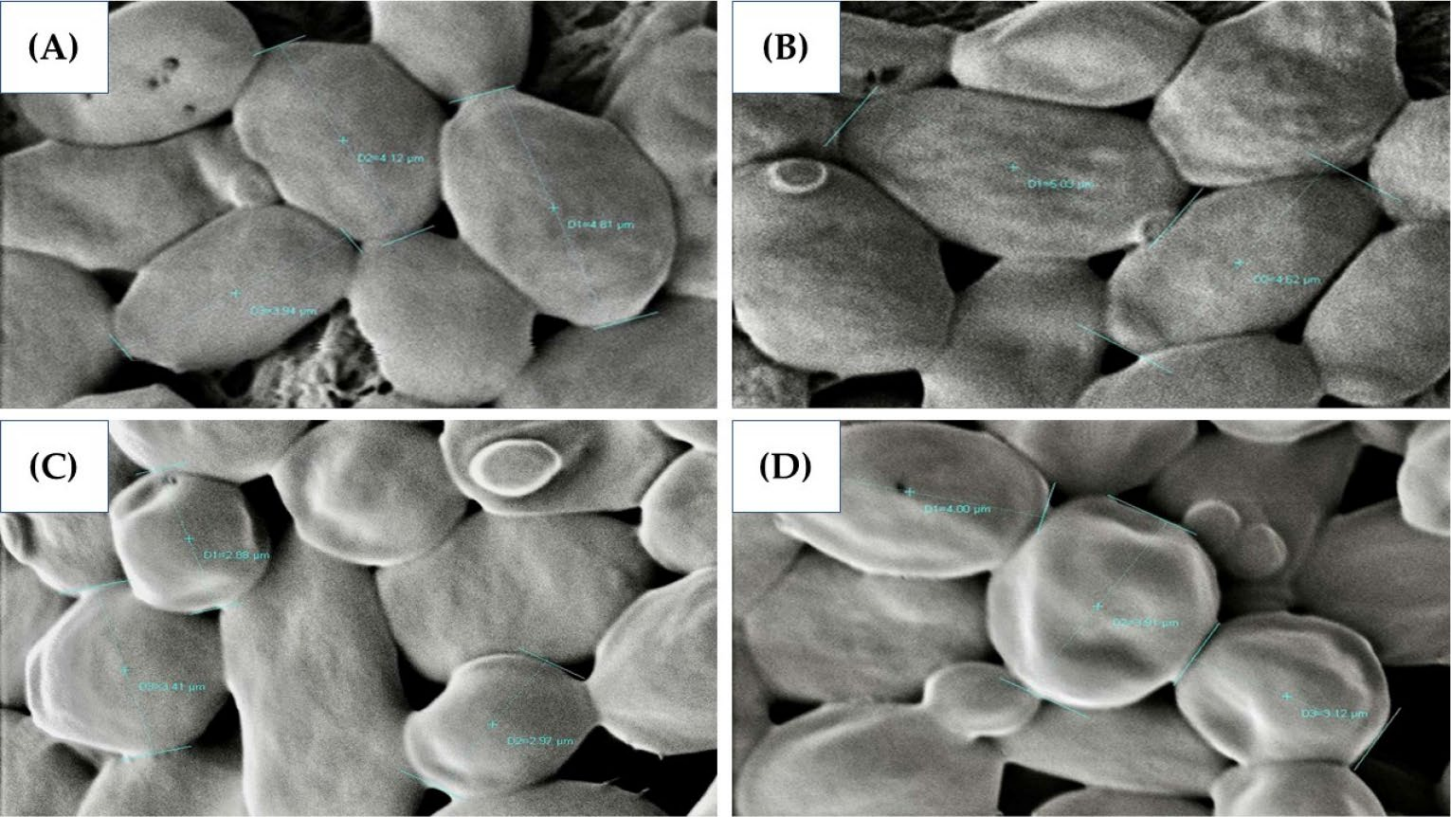
Cell morphology of the *P. kudriavzevii* parental (**A**) and evolved strains PkAC-7 (**B**), PkAC-8 (**C**), and PkAC-9 (**D**) visualized using SEM.

Figure 2 illustrates the cell morphology of the *P. kudriavzevii* parental and evolved strains using SEM. The parental strain exhibited a larger cell size (4.97 μm) and a smoother cell surface than the evolved strains. Increasing the concentrations of acetic acid in the culture medium led to a reduced yeast cell size. The evolved strain PkAC-9 displayed the smallest cell size (3.33 μm) compared to the others, i.e., 4.29 μm for PkAC-7 and 4.00 μm for PkAC-8. Furthermore, PkAC-9 also showed a rough cell surface compared to the others. The alteration of cell morphology in the *P. kudriavzevii* evolved strains, particularly cell size and cell surface, was possibly due to the adverse effect of high acetic acid concentrations during repetitive long-term cultivation. One of the cellular mechanisms proposed to be involved in the adaptation and tolerance to acetic acid is the remodeling of the yeast cell envelope^49^. High concentrations of acetic acid alter the molecular compositions and physical properties of the microbial plasma membrane and cell wall, causing a reduction in cell envelope permeability^49–52^, which is proposed to be essential for reducing acetic acid diffusion from the outside into the inside cellular structure. Ribeiro et al.^52^ demonstrated that adapting *S. cerevisiae* cells to acetic acid led to significant cell wall architecture alterations and increased cell wall stiffness, leading to higher acetic acid and lyticase activity resistance. Furthermore, yeast cell wall polysaccharide contents, specifically β-glucan and mannans, were also increased under acetic acid stress, making yeast cells more resistant to stress conditions. Regarding the morphological alteration of the *P. kudriavzevii* evolved strains, further studies should be performed, such as cell membrane and cell wall composition analysis.

### Growth characterization of the *P. kudriavzevii* parental and evolved strains under different stress conditions

**Figure 3:**
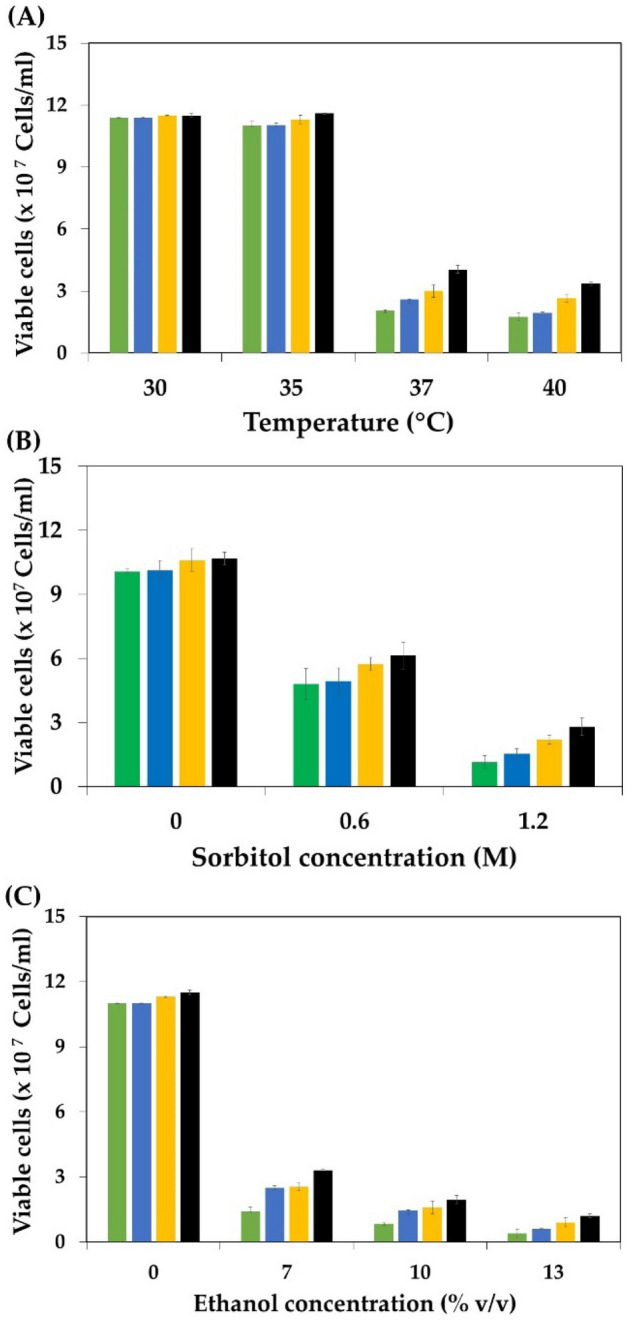
Effect of high temperature (**A**), osmotic (**B**), and ethanol (**C**) stress on the growth of the *P. kudriavzevii* parental (green box) and evolved strains PkAC-7 (blue box), PkAC-8 (yellow box), and PkAC-9 (black box) cultured at 35 °C.

As previously reported, the inhibitory effect of several stress conditions shares specific common characteristics, and resistance to one stress condition may lead to multiple stress tolerances^41,42^. Therefore, to check whether the selected evolved strains acquire multistress tolerance ability, the growth performance of the *P. kudriavzevii* parental and selected evolved strains under different stress conditions was evaluated and compared. The effect of high-temperature stress, which has been shown to inhibit microbial cell growth and cause cell damage resulting in cell death, was first evaluated. As shown in Fig. 3A, the growth of the *P. kudriavzevii* parental strain and all the evolved strains at 30 °C was similar. However, slightly higher growth of the evolved strains, particularly strains PkAC-8 and PkAC-9, occurred at 35 °C compared to the parental strain, which might be because these strains experienced a relatively high temperature of 35 °C during adaptation. The growth of the parental strain markedly decreased when the incubation temperature increased from 35 to 37 and 40 °C, in line with the results of Chamnipa et al.^38^ and Pilap et al.^42^, who reported a reduction in the cell viability of *P. kudriavzevii* under high-temperature conditions. Interestingly, all of the evolved strains exhibited significantly higher growth than the parental strain at 37 and 40 °C, suggesting that the evolved strains acquired thermotolerance ability during long-term adaptation in acetic acid. The acquisition of thermotolerance during long-term evolution adaptation to acetic acid or a mixture of lignocellulosic inhibitors, such as acetic acid, furfural, and vanillin, has also been reported in *S. cerevisiae*^37,44^ and *Kluyveromyces marxianus*^53^.

In addition to thermal stress, yeasts may also encounter other stress conditions during fermentation, particularly osmotic stress due to the high sugar concentration in the fermentation medium and ethanol stress due to ethanol accumulation during fermentation. These stresses have been shown to negatively affect microbial cell growth and viability, especially at relatively high temperatures^13,29^. Various concentrations of sorbitol as an osmotic stressor, including 0.6 and 1.2 M, were tested for their negative impact on yeast cell viability at 35 °C, and the results are illustrated in Fig. 3B. Although the *P. kudriavzevii* parental strain can grow in medium containing up to 1.2 M sorbitol, its growth was significantly lower than in the control medium without sorbitol supplementation and in the medium with 0.6 M sorbitol. All evolved strains exhibited higher growth than the parental strain; specifically, strain PkAC-9 displayed the highest growth under all osmotic stress conditions. As previously reported, high osmotic pressure due to high concentrations of sorbitol had negative effects on yeast cell growth, cell viability, and metabolic activity^47,54,55^. Notably, the high growth performance of all the evolved strains under high sorbitol concentrations indicates that all these strains may have acquired osmotolerance ability toward osmotic stress. Similar work has also been reported in the thermotolerant yeast *K. marxianus*, in which a thermal-adapted strain exhibited significant osmotolerance toward a high glucose concentration of 16% (w/v) at 40 °C^29^. Another study by Gurdo et al.^44^ also showed that the acetic acid-adapted strain of *S. cerevisiae* exhibited osmotolerance toward osmotic shock due to a high sorbitol concentration.

Ethanol is one of the primary end products of sugar metabolism in ethanologenic microorganisms and can become a significant stressor if high ethanol concentrations accumulate in the culture broth during fermentation. High levels of ethanol inhibit cell growth, cell viability, and metabolic activity, resulting in lowered ethanol yield and productivity^29,42,56^. Ultimately, ethanol stress also causes the modification of the plasma membrane and the denaturation of several macromolecules, such as DNA, RNA, and proteins, which can lead to cell death^57,58^. The inhibitory effect of ethanol stress on the growth of the *P. kudriavzevii* parental and evolved strains was monitored, and the results are presented in Fig. 3C. The growth was not significantly different when yeast cells were grown in medium without ethanol supplementation. A remarkable decrease in the growth of all yeast strains was observed when the ethanol concentrations in the culture medium increased to 7, 10, and 13% (v/v). The *P. kudriavzevii* evolved strains are more resistant to ethanol toxicity than the parental strain under all stress conditions, suggesting that these evolved strains acquired ethanol tolerance ability, similar to an acetic acid-adapted strain of *S. cerevisiae*^44^ and a thermal-adapted strain of *K. marxianus*^29^.

**Figure 4:**
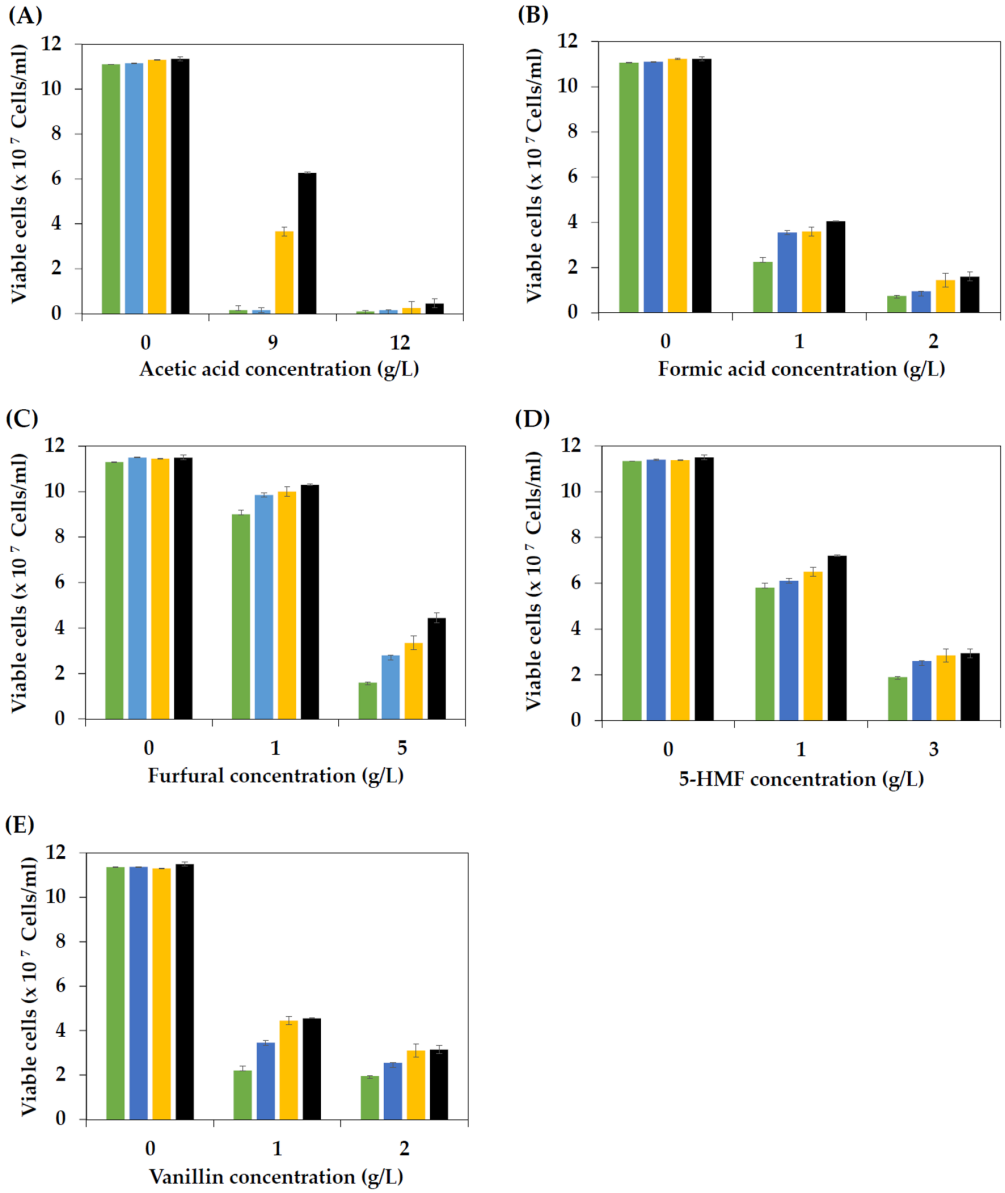
Effect of acetic acid (**A**), formic acid (**B**), furfural (**C**), 5-HMF (**D**), and vanillin (**E**) stress on the growth of the *P. kudriavzevii* parental (green box) and evolved strains PkAC-7 (blue box), PkAC-8 (yellow box), and PkAC-9 (black box) cultured at 35 °C for 48 h.

Acid or thermal pretreatment of lignocellulosic biomass generates fermentable sugars, such as glucose, xylose, and arabinose, as the major products, and some lignocellulosic inhibitors, including weak acids, furan derivatives, and phenolic compounds, as byproducts. These byproducts can inhibit microbial growth, metabolism, and fermentation activity in different manners depending on the types and concentrations of substances, fermentation conditions, and microbial species^42,59–62^. As reported in the literature, the acetic acid-adapted strain of *S. cerevisiae* and the thermal-adapted strain of *K. marxianus* exhibited multistress tolerance toward lignocellulosic inhibitors^29,44^. To check whether the evolved strains of *P. kudriavzevii* possess multistress tolerance properties against lignocellulosic inhibitors, the effect of individual and a mixture of lignocellulosic inhibitors, including acetic acid, formic acid, furfural, 5-HMF, and vanillin, on the growth of the *P. kudriavzevii* parental and evolved strains was evaluated. Regarding acetic acid, a byproduct from hemicellulose after releasing the acetate group, and formic acid, a product generated from the transformation of 5-HMF and furfural, low concentrations of these acids can suppress yeast cell growth. In addition, high concentrations of acetic and formic acids negatively inhibit transmembrane proton transport, interfere with pH homeostasis, and disturb cellular membrane structure and protein stability, leading to cell death^63,64^. The results in this study demonstrated that the growth of the *P. kudriavzevii* parental and evolved strains was similar when grown in medium without acetic acid supplementation. However, the growth of the evolved strains, particularly PkAC-8 and PkAC-9, was significantly higher than that of PkAC-7 and the parental strains when cultured at 35 °C in medium supplemented with 9 g/L acetic acid. Almost no growth of the parental strain was observed in medium containing 12 g/L acetic acid, similar to that reported in the wild-type strains of *P. kudriavzevii* (LF98, LF101, LF119, AC1, and AC4)^42^ and *S. cerevisiae* (Y8)^44^. Interestingly, the evolved strain PkAC-9 can grow better than the other strains under this stress condition (Fig. 4A), possibly correlated with the long-term exposure to acetic acid during the adaptation process. Similar growth patterns of the parental and evolved strains were observed in the medium supplemented with formic acid at different concentrations. A remarkable decrease in the growth of all yeast strains occurred when cultured in medium supplemented with 1 and 2 g/L formic acid. Notably, the parental strain was more sensitive to formic acid than the evolved strains since its growth was lower than that of the evolved strains under all formic acid stresses. Among the selected evolved strains, PkAC-9 exhibited higher growth than PkAC-7 and PkAC-8 in the medium supplemented with 2 g/L formic acid (Fig.4B). These findings conclude that the evolved *P. kudriavzevii* strains were more resistant to weak acids in the lignocellulosic hydrolysate than the parental strain, similar to results reported in the literature^29,44,53^.

Furfural and 5-HMF, the furan derivatives generated from the degradation of pentose and hexose sugars, respectively, are known to inhibit yeast cell growth even at low concentrations (0.5–2.0 g/L). Several macromolecules, such as DNA, RNA, proteins, chromatin, vacuoles, mitochondria, and cell membranes, can be damaged in the presence of these stress compounds^41,65^. Additionally, they have also been shown to induce the accumulation of reactive oxygen species (ROS) in yeast cells, causing oxidative stress, and reducing the activity of several enzymes involved in glycolysis and ethanol production pathways, such as pyruvate dehydrogenase, alcohol dehydrogenase, and aldehyde dehydrogenase^66,67^. The effect of furfural and 5-HMF on the growth of the *P. kudriavzevii* parental and evolved strains was tested using YM medium containing 0, 1, and 5 g/L furfural and 0, 1, and 3 g/L 5-HMF. In the medium without furfural, the growth of the *P. kudriavzevii* parental and evolved strains was similar. A slight decrease in the growth of all yeast strains was observed when the medium was supplemented with 1 g/L furfural. However, a significant decrease in the growth of all yeast strains occurred when the furfural concentration in the medium increased to 5 g/L. The parental strain displayed the lowest growth, while PkAC-9 yielded the highest growth under all furfural stress treatments (Fig. 4C). Considering the inhibitory effect of 5-HMF on the growth of yeast cells, 1 and 3 g/L of 5-HMF caused a remarkable decrease in the growth of all of the yeast strains. The parental *P. kudriavzevii* strain was more sensitive to 5-HMF than the evolved strains. Again, the evolved strain PkAC-9 exhibited the highest growth in the medium containing 1 and 3 g/L 5-HMF, although its growth was not significantly different from that of PkAC-8 in the medium containing 3 g/L 5-HMF (Fig. 4D). These results indicated that the evolved *P. kudriavzevii* strains acquired multistress tolerance toward furan derivatives generated during the lignocellulosic pretreatment process.

In addition to weak acids and furan derivatives, phenolic compounds such as vanillin have also been shown to inhibit cell growth and metabolic activity. Vanillin, a degradation product of guaiacylpropane units of lignin, can inhibit yeast cell growth and fermentation activity at very low concentrations, making it one of the most effective inhibitors in lignocellulosic hydrolysates. It negatively impacts the structure and integrity of yeast cell membranes^68^ and the fermentation efficiency of yeast cells. A recent study by Pattanakittivorakul et al.^29^ demonstrated that vanillin at 1 g/L inhibits the growth and ethanol production performance of the *Kluyveromyces marxianus* wild-type strain, a thermotolerant yeast for high-temperature ethanol production. The adverse effect of vanillin on the growth of the *P. kudriavzevii* parental and evolved strains was evaluated, and the results are summarized in Fig. 4E. Vanillin at 1 and 2 g/L caused a significant reduction in yeast cell growth. The growth inhibition of vanillin was more pronounced in the parental strain than in the evolved strains. The evolved strains PkAC-8 and PkAC-9 exhibited the highest growth in medium supplemented with 1 and 2 g/L vanillin. These results were similar to those reported in *K. marxianus*^29,53^.

Based on the growth properties under different stress conditions, acquiring multistress tolerance may evolve in *P. kudriavzevii* during long-term adaptation to acetic acid. In particular, the evolved strain PkAC-9 displayed superior multistress tolerance toward heat, ethanol, osmotic, and lignocellulosic inhibitors, making it a robust yeast strain for second-generation bioethanol production.

**Figure 5:**
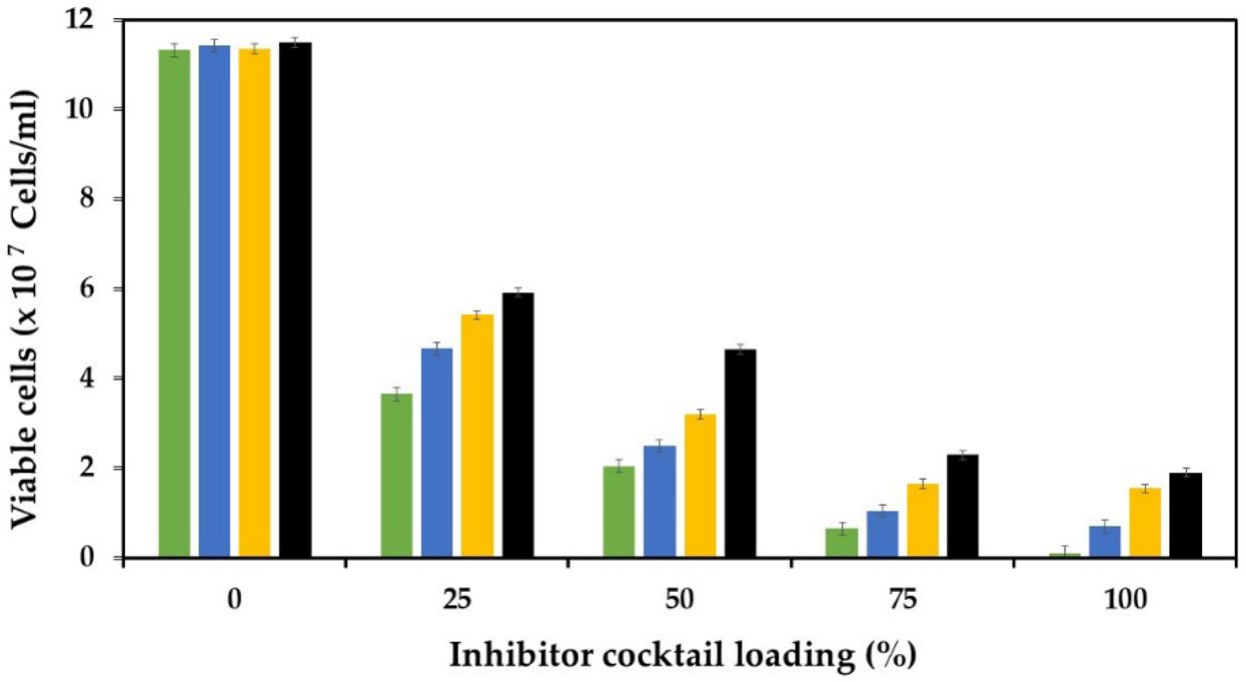
Effect of inhibitor cocktail at different loadings on the growth of the *P. kudriavzevii* parental (green box) and evolved strains PkAC-7 (blue box), PkAC-8 (yellow box), and PkAC-9 (black box) cultivated at 35 °C for 48 h.

Considering that the lignocellulosic pretreatment process often generates a complex mixture of inhibitors, the synergistic effects of several inhibitors in lignocellulosic hydrolysate on the microbial growth, metabolic activity, and fermentation efficiency of yeast cells have also been reported^29,42,53,69^. Thus, the inhibitory effect of multiple inhibitors, including acetic acid, formic acid, furfural, 5-HMF, and vanillin, on the growth of the *P. kudriavzevii* parental and evolved strains was examined (Fig. 5). Different loadings of the inhibitor cocktail were tested, and the lowest growth of all of the yeast strains occurred at 100% inhibitor loading, suggesting that this stress condition was more toxic to yeast cells. The *P. kudriavzevii* parental strain was more susceptible to the cocktail inhibitor than the evolved strains. When the concentration of the inhibitor cocktail was reduced (75, 50, and 25% loading), the growth of the yeast cells was increased. The highest growth occurred in the evolved strain PkAC-9 under all inhibitor loading conditions, suggesting that this strain is more resistant to multiple stresses than the others, similar to the results observed in the experiments using a single stress condition.

### Ethanol production from SBH by the *P. kudriavzevii* parental and evolved strains

Sugarcane bagasse, a byproduct of sugarcane manufacturing, is one of the least expensive and most significant readily available feedstocks in Thailand. In 2022, Thailand was the world’s fourth-largest sugarcane-producing country, producing approximately 10.3 million tonnes of sugar. Forty-seven sugar factories are in operation in Thailand, generating approximately 20 million metric tonnes of sugarcane bagasse annually^70^. This byproduct contains approximately (on a dry weight basis) 36–40% cellulose, 28–32% hemicellulose, and 12–14% lignin^71^, making it one of the highest-potential feedstocks for second-generation bioethanol production. The sugarcane bagasse used in the present study contained 49.09% cellulose, 29.30% hemicellulose, and 9.74% lignin on a dry weight basis, slightly different from values reported in the literature. For instance, 39.8% cellulose, 28.6% hemicellulose, and 22.5% lignin were reported by Oliveira et al.^72^, while 39.2% cellulose, 37.9% hemicellulose, and 11.8% lignin were reported by de Souza et al.^73^. Cellulose, hemicellulose, and lignin contents of 38.6%, 27.9%, and 17.8% on a dry basis, respectively, were also reported^74^, and most recently, Chamnipa et al.^75^ reported cellulose, hemicellulose, and lignin contents in sugarcane bagasse with values of 47.9%, 31.4%, and 10.3% on a dry basis, respectively. Since several factors, such as plant varieties, environmental growth conditions, cultivation areas, harvesting, and manufacturing processes, affect plant growth and physiological properties and the structure of plant biomass, the differences in the chemical compositions of sugarcane bagasse may be attributed to such factors^76^.

Although several lignocellulosic pretreatment processes have been reported, the chemical process using dilute acid is typically used since it has high efficiency in separating cell wall components, yielding high concentrations of fermentable sugars. It is also a simple process with a low-cost operation that can be adopted for wide-scale applications compared to physicochemical and biological processes^78–80^. In this study, dilute acid pretreatment of sugarcane bagasse using 3% (v/v) sulfuric acid at 121 °C and 15 psi for 60 min was performed, yielding a liquid fraction called acid hydrolysate, as mentioned in the Materials and Methods section. This acid hydrolysate contained 25.81 g/L of total sugar, in which xylose was the most predominant sugar, accounting for 20.32 g/L, followed by arabinose and glucose at concentrations of 2.90 and 2.59 g/L, respectively. Acetic acid was the most predominant lignocellulosic inhibitor in the acid hydrolysate, accounting for 3.77 g/L. Other inhibitors found in this hydrolysate included formic acid (0.26 g/L), furfural (0.08 g/L), 5-HMF (0.46 g/L), and vanillin (0.03 g/L).

The solid fraction after acid pretreatment was hydrolyzed using cellulase, and the resulting hydrolysate (enzymatic hydrolysate) was collected and analyzed for its chemical composition. As a result, a total sugar content of 63.88 g/L was obtained, which was approximately 2.5-fold higher than that in the acid hydrolysate. Glucose, a major product of cellulose hydrolysis, was the most abundant sugar found in the hydrolysate, comprising 43.54 g/L. Xylose and arabinose were also detected at concentrations of 19.29 and 1.05 g/L, slightly lower than that of the acid hydrolysate. All of the inhibitors found in the acid hydrolysate, except vanillin, were also present in the enzymatic hydrolysate, i.e., acetic acid (0.89 g/L), formic acid (2.80 g/L), furfural (1.57 g/L), and 5-HMF (0.23 g/L).

One of the aims of this study is to use undetoxified acid hydrolysate as feedstock for ethanol production. Therefore, ethanol production by the *P. kudriavzevii* parental and evolved strains using the acid hydrolysate as a primary feedstock was performed. Unfortunately, very low yeast cell growth and ethanol production were detected after 72 h of fermentation at 35 °C, probably due to the toxic effect of sulfuric acid and a low sugar level, mainly glucose, in the hydrolysate. Thus, a mixture of acid and enzymatic hydrolysates at a ratio of 1:1 was used as a feedstock instead of using acid hydrolysate alone. As a result, all yeast strains can grow and produce ethanol in different manners, possibly due to a reduction in sulfuric acid toxicity and an increase in the glucose concentration in the hydrolysate. Indeed, a mixture of acid and enzymatic hydrolysate contained 30.06 g/L of total sugar, in which glucose and xylose were the most predominant sugars, comprising 17.44 and 11.18 g/L, respectively. The concentrations of inhibitors in the acid hydrolysate were reduced from 3.77 to 1.90 g/L for acetic acid, 0.26–0.12 g/L for formic acid, 0.08–0.03 g/L for furfural, and 0.46–0.15 g/L for 5-HMF. Vanillin was not detected in the hydrolysate mixture.

The time profile of ethanol production from a mixture of acid and enzymatic hydrolysate of sugarcane bagasse by *P. kudriavzevii* parental and evolved strains was shown in Supplementary Fig. S2. The sugar utilization by the parental strain was lower than that of the evolved strains. PkAC-9 displayed the highest sugar utilization than the others. The maximum ethanol concentrations produced by different yeast strains were detected at 72 h after fermentation. The kinetic parameters of the ethanol production by *P. kudriavzevii* parental and evolved strains are summarized in Table 2. The results revealed that all evolved strains produced higher ethanol concentrations, productivity, and yields than the parental strain. The *P. kudriavzevii* parental strain produced the lowest ethanol concentration of 6.60 g/L, with an ethanol yield of 0.33 g/g, while strain PkAC-9 produced the highest ethanol content of 11.02 g/L and ethanol yield of 0.50 g/g. Notably, all of the evolved strains consumed sugar in the SBH better than that of the parental strain, specifically strain PkAC-9 consumed approximately 16% higher sugar than the parental strain (Table 2). The present results align with the findings of Pattanakittivorakul et al.^29^, who demonstrated that the thermal-adapted strain of *K. marxianus* assimilated greater glucose content and produced higher ethanol concentration than the parental strain. As previously discussed, remodeling of yeast cell envelop^49^ or alteration of yeast cell wall and cell wall components^52^ triggered by acetic acid stress may protect proteins or enzymes involved in the ethanol production pathway, leading to high sugar conversion and ethanol fermentation efficiency.

**Table 2:** Ethanol production by the *P. kudriavzevii* parental and evolved strains at 35 °C using a mixture of acid and enzymatic hydrolysate of sugarcane bagasse as feedstock.

Yeast strain	Fermentation parameter			
	P (g/L)	Qp (g/L.h)	Yp/s (g/g)	TS (%)
Wild-type	6.60 ± 0.02^d^	0.10 ± 0.00^d^	0.33 ± 0.01^c^	66.73 ± 0.13^d^
PkAC-7	8.41 ± 0.02^c^	0.12 ± 0.00^c^	0.46 ± 0.01^b^	70.06 ± 0.08^c^
PkAC-8	9.75 ± 0.03^b^	0.14 ± 0.00^b^	0.48 ± 0.02^ab^	73.38 ± 0.10^b^
PkAC-9	11.02 ± 0.03^a^	0.15 ± 0.00^a^	0.50 ± 0.01^a^	77.53 ± 0.02^a^

P: ethanol concentration (g/L); Qp: ethanol productivity (g/L h); Yp/s: ethanol yield (g ethanol produced/g total sugar consumed); TS: total sugar consumed (%). Means ± SDs with the same letters in the same column are not significantly different at *p* ≤ 0.05 based on DMRT analysis.

It has been previously reported that *P. kudriavzevii* could produce a high level of ethanol from lignocellulosic hydrolysate. For instance, a maximum ethanol concentration of 35.51 g/L was produced from enzymatic hydrolysate of sugarcane bagasse containing 85 g/L glucose at 37 °C^38^. Phong et al.^39^ reported a maximum ethanol concentration of 36.91 g/L from dilute acid hydrolysate of pineapple waste containing 103 g/L total sugar at 45 °C. Recently, Hoppert et al.^80^ demonstrated that *P. kudriavzevii* HYPK213_ELA could produce a maximum ethanol concentration of 56.8 g/L from wheat straw hydrolysate at 37% w/w solid loading at 40 °C. Regarding the ethanol production in the current study, a low ethanol concentration obtained from a mixture of acid and enzymatic hydrolysate of sugarcane bagasse by *P. kudriavzevii* evolved strains may be due to a low sugar concentration in the fermentation medium. Further study on ethanol production from SBH containing high sugar concentrations should be performed, and it is currently under investigation.

In comparison with other studies using SBH as feedstock, the ethanol concentration, productivity, and yield produced by *P. kudriavzevii* PkAC-9 were higher than those reported in the literature, such as *Scheffersomyces stipitis* UFMG-IMH-43.2^81^, *Spathaspora passalidarum* UFMG-HMD-1.1 and UFMG-HMD-14.1^82^, *Pichia* BY2^83^, and *P. stipitis* JCM 10742^10^. Based on the growth performance under different stress conditions, including heat, ethanol, osmotic, and lignocellulosic inhibitors stresses, together with the ethanol production efficiency, the evolved strains, particularly PkAC-9, are one of the most promising yeasts for ethanol production using undetoxified lignocellulosic hydrolysate as feedstock, which could eliminate the detoxification process and reduce the operating cost, making commercial lignocellulosic bioethanol production more sustainable.

Regarding the biological fermentation process, not only the microbial species but also the environmental fermentation conditions significantly impact ethanol yield and productivity. Several fermentation parameters, such as microbial cell concentration^84,85^, sugar concentration^47,84^, pH of the fermentation medium^85–87^, nitrogen sources^46,47,85^, and micro- and macronutrients^85,86^, have been shown to significantly influence ethanol production by yeast cells. Therefore, optimization conditions for ethanol production from lignocellulosic biomass employing a one-factor-at-a-time or a statistical experimental model using the Box‒Behnken design (BBD) or central composite design (CCD) based on the response surface methodology (RSM) should be performed to improve the fermentation efficiency.

Several cellular pathways are responsible for acetic acid stress in yeast cells. These pathways include cellular transport, pH homeostasis, metabolism, and stress-signaling pathways, in which many genes or proteins are involved in each pathway^87^. For instance, the aquaglyceroporin Fps1, Jen1, and Ady2 transporters proteins and the ABC transporter Pdr18 have been reported to play a crucial role in cellular transportation of acetic acid as well as lipid composition and cell membrane permeability (Mollapour and Piper^88^; Casal et al.^89^; Godinho et al.^90^). The plasma membrane H^+^-ATPase (Pma1) and vacuolar ATPase assembly protein (VMA3) have been shown to contribute to acetic acid stress resistance, which is part of the pH homeostasis of yeast cells (Ullah et al.^91^; Konarzewska et al.^92^). Regarding the metabolism and stress-signaling pathways, several genes and proteins related to carbohydrate metabolism, protein folding, lipid metabolism, amino acid metabolism, cell wall function, and transport are involved in acetic acid tolerance (Mira et al.^93^; Wang et al.^94^). Of these, the transcription factors Haa1p and Hog1 are the significant players contributing to acetic acid resistance in yeast cells (Mira et al.^95^; Kim et al.^96^).

Based on a literature review, the molecular mechanisms involved in multistress tolerance are complicated, involving several genes or proteins. For instance, three genes, *ICL1* (encoding isocitrate lyase), *CIT3* (encoding citrate synthase 3), and *ADY2* (encoding acetate transporter protein), are upregulated upon heat stress at 45 °C and are proposed to be involved in acetic acid tolerance in *K. marxianus*^29^. In *S. cerevisiae*, tolerance to acetic acid is also correlated with the expression of these three genes^97,98^ as well as other genes, including *ASG1* (encoding transcriptional regulator), *ADH3* (encoding alcohol dehydrogenase), *SKS1* (encoding protein kinase), and *GIS4* (encoding a protein involved in ion homeostasis)^48^, while the ability to withstand oxidative stress is associated with the activity of antioxidative enzymes, specifically catalase (CAT) and glutathione S-transferase (GST)^44^. The synthesis of some metabolites in response to heat stress, such as trehalose and glycogen, also improved the thermotolerance and ethanol fermentability of *S. cerevisiae*^37^. The acquisition of thermotolerance and fermentation efficiency of *S. cerevisiae* are also associated with genes encoding the proteins involved in the ethanol production pathway, DNA repair, and oxidative stress^85^. Genes for ergosterol biosynthesis are required for tolerance to vanillin stress^99^, while several genes involved in ionic homeostasis, heat protection, trehalose synthesis, antioxidant defense, and ATP production have been shown to be involved in ethanol stress in *S. cerevisiae*^100^. A recent study by Rahman et al.^101^ demonstrated that the response of *P. kudriavzevii* toward acetic acid includes the activation of genes involved in the tricarboxylic acid cycle, ATP-binding cassette transporters, and protein folding, sorting and degradation. Thus, it could be concluded from this information that different organisms employ different mechanisms to deal with different stress conditions. Considering the molecular mechanism involved in multistress tolerance in *P. kudriavzevii* evolved strains, further studies such as transcriptomic, proteomic, and metabolomic analyses or whole-genome sequencing should be performed.

## Conclusions

Based on the repetitive long-term cultivation in acetic acid at 35 °C, three potential multiple-stress-tolerant strains of *P. kudriavzevii*, namely, PkAC-7, PkAC-8, and PkAC-9, were obtained. Although their cell size and cell surface were slightly different, all of them exhibited multistress tolerance toward heat, ethanol, osmotic, and lignocellulosic inhibitors, including acetic acid, formic acid, furfural, 5-HMF, and vanillin, individually and in a mixture of inhibitor cocktails. Furthermore, the evolved strains, particularly strain PkAC-9, produced the highest ethanol concentration, productivity, and yield from undetoxified SBH. This study provides evidence that ALE using acetic acid as a selective pressure is an effective method to improve the multistress tolerance of *P. kudriavzevii* for industrial ethanol production using lignocellulosic biomass as a feedstock.

### Supplementary Information

Supplementary Figures.

## Data Availability

All data generated or analyzed during this study are included in this published article, and Supplementary Information files associated with this article can be found in the online version.
