# Supplementary material for: Adaptive laboratory evolution under acetic acid stress enhances the multistress tolerance and ethanol production efficiency of Pichia kudriavzevii from lignocellulosic biomass
Source: Sci Rep. 2023 Nov 28;13:21000. doi: 10.1038/s41598-023-48408-7 (PMC10684600; doi:10.1038/s41598-023-48408-7)
Supplement: Supplementary file 1 — Supplementary Figures. [file 41598_2023_48408_MOESM1_ESM.docx]

*Research article*:

**Adaptive laboratory evolution under acetic acid stress enhances the multistress tolerance and ethanol production efficiency of *Pichia kudriavzevii* from lignocellulosic biomass**

Sureeporn Dolpatcha^1^, Huynh Xuan Phong^2^, Sudarat Thanonkeo^3^, Preekamol Klanrit^1,4^, Mamoru Yamada^5,6^ and Pornthap Thanonkeo^1,4,*^

^1^ Department of Biotechnology, Faculty of Technology, Khon Kaen University, Khon Kaen 40002, Thailand; [sureeporndo@kkumail.com](mailto:sureeporndo@kkumail.com) (S.D.); kpreek@kku.ac.th (P.K.); portha@kku.ac.th (P.T.)

^2^ Department of Microbiology Biotechnology, Biotechnology Research and Development Institute, Can Tho University, Can Tho 900000, Vietnam; [hxphong@ctu.edu.vn](mailto:hxphong@ctu.edu.vn) (H.X.P.)

^3^ Walai Rukhavej Botanical Research Institute, Mahasarakham University, Maha Sarakham 44150, Thailand; sudarat.t@msu.ac.th

^4^ Fermentation Research Center for Value Added Agricultural Products (FerVAAPs), Faculty of Technology, Khon Kaen University, Khon Kaen 40002, Thailand

^5^ Department of Biological Chemistry, Faculty of Agriculture, Yamaguchi University, Yamaguchi 753-8515, Japan; m-yamada@yamaguchi-u.ac.jp

^6^ Research Center for Thermotolerant Microbial Resources, Yamaguchi University, Yamaguchi 753-8515, Japan

***** Correspondence: Pornthap Thanonkeo

Department of Biotechnology, Faculty of Technology, Khon Kaen University, Khon Kaen 40002, Thailand; e-mail address: [portha@kku.ac.th](mailto:portha@kku.ac.th); Tel.: +66-819743340

**Supplement Figure**


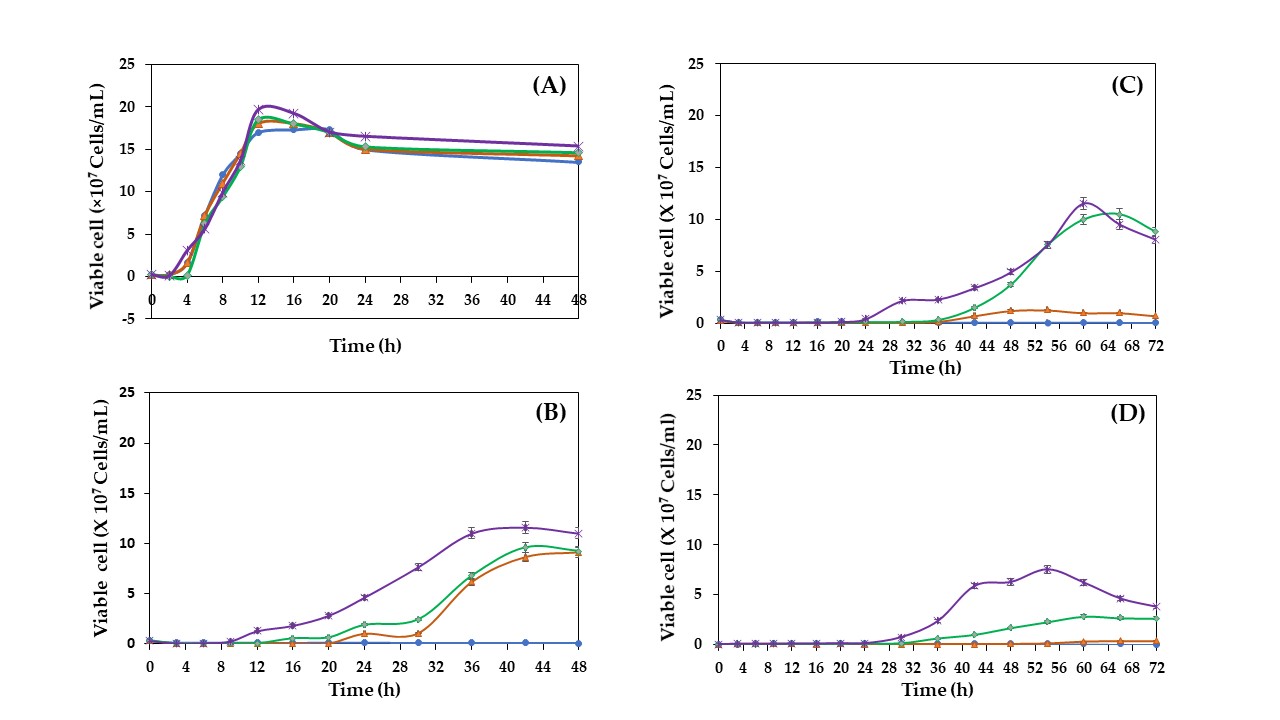


**Figure S1.** Growth of *Pichia kudriavzevii* wild-type (●), and evolved strain PkAC-7 (▲), PkAC-8 (♦), and PkAC-9 (⁎) in YM medium without acetic acid (A), and with acetic acid supplementation at 7 g/L (B), 8 g/L (C), and 9 g/L (D).

**Figure S2.** Time course of ethanol production from a mixture of acid and enzymatic hydrolysate of sugarcane bagasse by *Pichia kudriavzevii* wild-type and evolved strains at 35 °C. Symbols: ●, wild-type; ▲, PkAC-7; ♦, PkAC-8; ■, PkAC-9. Solid lines are ethanol concentration, and dash lines are total sugar concentration.
